# Breaking the Rules: Museum Data Reveal Divergent Support for Classic Ecogeographical Rules in North American Flying Squirrels

**DOI:** 10.1002/ece3.74470

**Published:** 2026-10-12

**Authors:** Tiffany A. Paalman, Joseph P. Morton, Cori Lopazanski, Brian R. Silliman, Amy T. Wolf

**Affiliations:** ^1^ Department of Natural and Applied Sciences University of Wisconsin–Green Bay Green Bay Wisconsin USA; ^2^ Division of Marine Science and Conservation, Nicholas School of the Environment Duke University Beaufort North Carolina USA

**Keywords:** Allen's rule, Bergmann's rule, flying squirrel, *Glaucomys*, morphology, museum data, North America

## Abstract

Ecogeographic generalizations or “rules”, such as Bergmann's rule and Allen's rule, propose that endotherms in colder climates evolve larger bodies and shorter appendages to minimize heat loss. These patterns are hypothesized to reflect physiological adaptations to thermal stress through reduced surface area‐to‐volume ratios. Although these rules have been extensively evaluated across birds and mammals, their applicability to arboreal gliders remains untested, with little consideration of how alternative ecological and evolutionary drivers may structure morphological variation in flying squirrels across environmental gradients. Using digitized museum specimens of North American flying squirrels (*Glaucomys sabrinus, Glaucomys oregonensis*, and *Glaucomys volans*), we tested these classic ecogeographic principles by examining how weight, total length, tail length, and ear length vary across a latitudinal gradient. We found significant support for Bergmann's rule, with body mass and total length increasing with latitude. In contrast, patterns in appendage morphology provided limited support for Allen's rule. Ear length exhibited a weak positive relationship with latitude, while tail length strongly increased with latitude, contradicting the prediction that appendages become shorter in colder climates. These findings suggest that thermoregulatory selection alone cannot explain morphological variation in North American flying squirrels and that functional demands, particularly those associated with gliding performance, may play a stronger role in shaping tail morphology. Overall, our results demonstrate that although classic ecogeographic rules provide a useful framework for understanding morphological variation, they do not fully capture the multiple selective pressures acting on *Glaucomys* species.

## Graphical abstract

Using digitized museum specimens of North American flying squirrels ( *Glaucomys sabrinus* , *Glaucomys oregonensis*, and *Glaucomys volans* ), we documented mixed support of classic ecogeographic rules, Bergmann's rule and Allen's rule, by examining how weight, total length, tail length, and ear length varied across a latitudinal gradient. Analyses did not support Allen's rule for tail length, which was positively correlated with latitude. This result suggests that thermoregulatory‐adapted pressures compete with aerodynamic demands of the tail for glide performance.

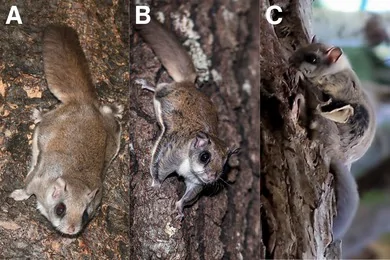

## 1 Introduction

Ecogeographic rules that describe how animal morphology varies with climatic gradients offer a powerful framework for understanding organismal adaptation, thermoregulation, and biogeographic patterning in endotherms (Gaston et al. 2008; Mayr 1956). Two foundational ecogeographical rules, Bergmann's rule and Allen's rule, respectively describe patterns of body size and appendage length across thermal gradients (Allen 1877; Bergmann 1847). Bergmann's rule posits that within a species, individuals in colder, higher latitude environments tend to exhibit larger body sizes (Bergmann 1847), while Allen's rule predicts that individuals at higher latitudes should have shorter appendages (Allen 1877). These morphological trends are considered thermal adaptations, where a compact body form with a lower surface‐area‐to‐volume ratio reduces energetic costs of thermoregulation in cold climates (Blackburn et al. 1999; Tilkens et al. 2007). The thermoregulatory‐adapted predictions of Bergmann and Allen's rules have broad support across endothermic taxa (McQueen et al. 2022; Brown and Lee 1969; Ballinger and Nachman 2022); however, there are documented taxon‐ and trait‐specific deviations which suggest that for endothermic taxa, other drivers may be more constraining than thermoregulation (He et al. 2023). For example, a negative relationship between wing length over temperature gradients in a widespread Andean duck species indicates a deviation from Allen's rule, suggesting that aerodynamic and physiological constraints may override expected environmental patterns (Gutiérrez‐Pinto et al. 2014). Identifying alternative drivers, such as these, and which species they affect, could help explain exceptions to these general rules and better predict organismal responses to environmental conditions (Alhajeri et al. 2020; Gaston et al. 2008; Meiri and Dayan 2003). Here, we use North American flying squirrel species, the northern flying squirrel ( *Glaucomys sabrinus* ), Humboldt's flying squirrel (*Glaucomys oregonensis*), and the southern flying squirrel ( *Glaucomys volans* ), as a model species group to test the relationship between latitude and body size (mass and length) and appendage length (ear and tail length).

*Glaucomys* squirrels are a widespread resident of forests throughout North America (Weigl 2007; Gilmore and Gates 1985; Winchell et al. 2024) (Figure 1). Across their ranges, flying squirrels experience substantial variation in temperature, seasonality, and forest structure, making this species group well‐suited for testing ecogeographic rules that predict morphological clines along latitudinal or thermal axes. While existing literature addresses morphological adaptations for flight correlated with environmental drivers in avian species, little evidence exists for ecogeographical relationships in gliding species. As a volant species, flying squirrels have key morphological traits (e.g., tail, patagium flaps, and limbs) that are critical for gliding and maneuvering within arboreal habitats (Bahlman et al. 2013). Specifically in *Glaucomys* spp., the tail comprises a substantial percentage of total ventral surface area (20%–30%, compared to 15%–20% in other arboreal squirrels), underscoring its importance in steering and stability (Thorington and Heaney 1981). Consequently, selection for gliding performance could override or weaken climate‐driven morphological adaptation, leading to patterns inconsistent with classic ecogeographic rules. When aerodynamic performance exhibits selective pressure, we predict that body length, body mass, and ear length conform to Bergmann's and Allen's rules, respectively, whereas tail length exhibits weaker conformity for *Glaucomys* species.

**FIGURE 1:**
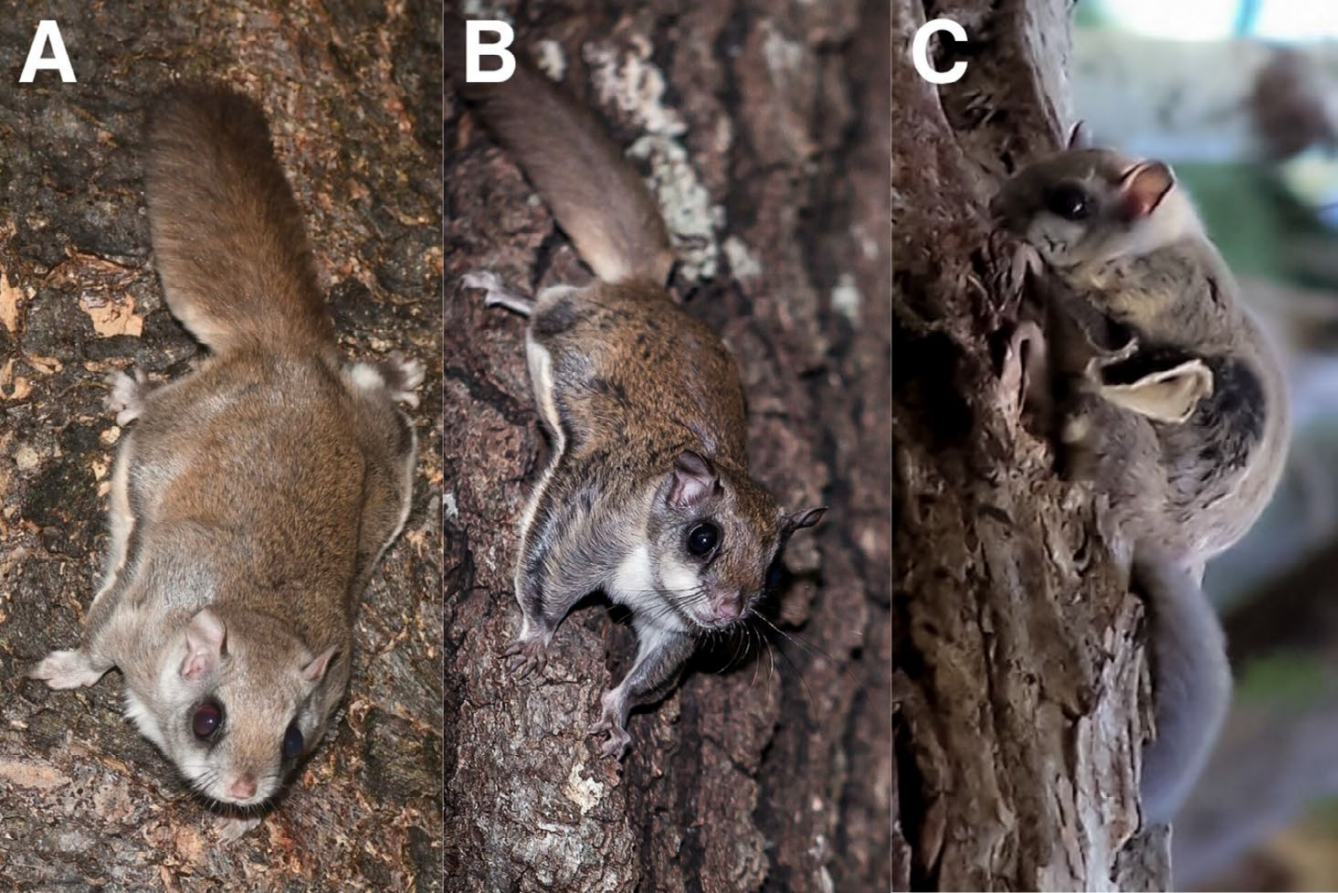
North American flying squirrels (genus *Glaucomys*). (A) Northern flying squirrel ( *G. sabrinus* ; photo: Henrique Pacheco). (B) Humboldt's flying squirrel ( *G. oregonensis* ; photo: Barbogast). (C) Southern flying squirrel ( *G. volans* ; photo: MimiMiaPhotography). All images sourced from Wikimedia Commons under Creative Commons license.

## 2 Methods

To assess the ecogeographical relationships across *Glaucomys* species, we leveraged morphological data from museum collections to investigate the relationship between latitude and body size (mass and length) and appendage length (ear and tail length). We compiled morphological trait data for 1649 *Glaucomys* specimens from publicly available museum databases and institutional collections, including Arctos (Arctos 2025), VertNet (VertNet 2024), the Smithsonian National Museum of Natural History Division of Mammals Collections (Smithsonian National Museum of Natural History 2024), and the Richter Museum of Natural History. To account for recent advances in *Glaucomys* genetic data (Arbogast et al. 2017), any *G. sabrinus* records shown to be within California, Oregon, or Washington were reclassified as *G. oregonensis* . Compiled data resulted in 814 *G. sabrinus* specimens, 475 *G. oregonensis* specimens, and 360 *G. volans* specimens. When available, data obtained from Arctos, VertNet, and the Smithsonian Collections included the date of collection, collection site with coordinates, sex, weight, total body length, tail length, and ear length. When a collection site was included in the record, but no coordinates were provided, coordinates were assigned using the central point of the most precisely indicated place. Manual evaluation of total body length, tail length, and ear length was conducted for specimens housed at the Richter Museum of Natural History (*n* = 4). Date of collection, collection site with coordinates, and sex were recorded according to the corresponding tag with each specimen. Specimens were laid flat, ventral‐side down over a measuring tape, with all measurements recorded to the nearest mm. Total body length was measured from the nose tip to the fleshy tip of the extended tail, excluding any hairs projecting beyond the tip. Tail length was measured from the tail base to the fleshy tip of the tail, excluding any hairs projecting beyond the tip. Ear length was measured from notch, lowest point of the ear opening, to tip of the ear.

First, we evaluated (1) whether weight and total body length conform to Bergmann's rule, (2) whether ear length conforms to Allen's rule, and (3) whether tail length exhibits reduced conformity to these thermoregulatory expectations across all species. We then repeated these analyses for each species individually. For all analyses, we used linear regression to test whether these morphological traits (total length, weight, ear length, and tail length) varied directionally with latitude.

All analyses were conducted in R (v 4.5.0). Specimens originated from 711 distinct collection sites spanning 48° of latitude (17.6° N to 66.3° N) (Figure 2) and collection years 1890 to 2023, providing broad spatial and temporal coverage for testing ecogeographic hypotheses.

**FIGURE 2:**
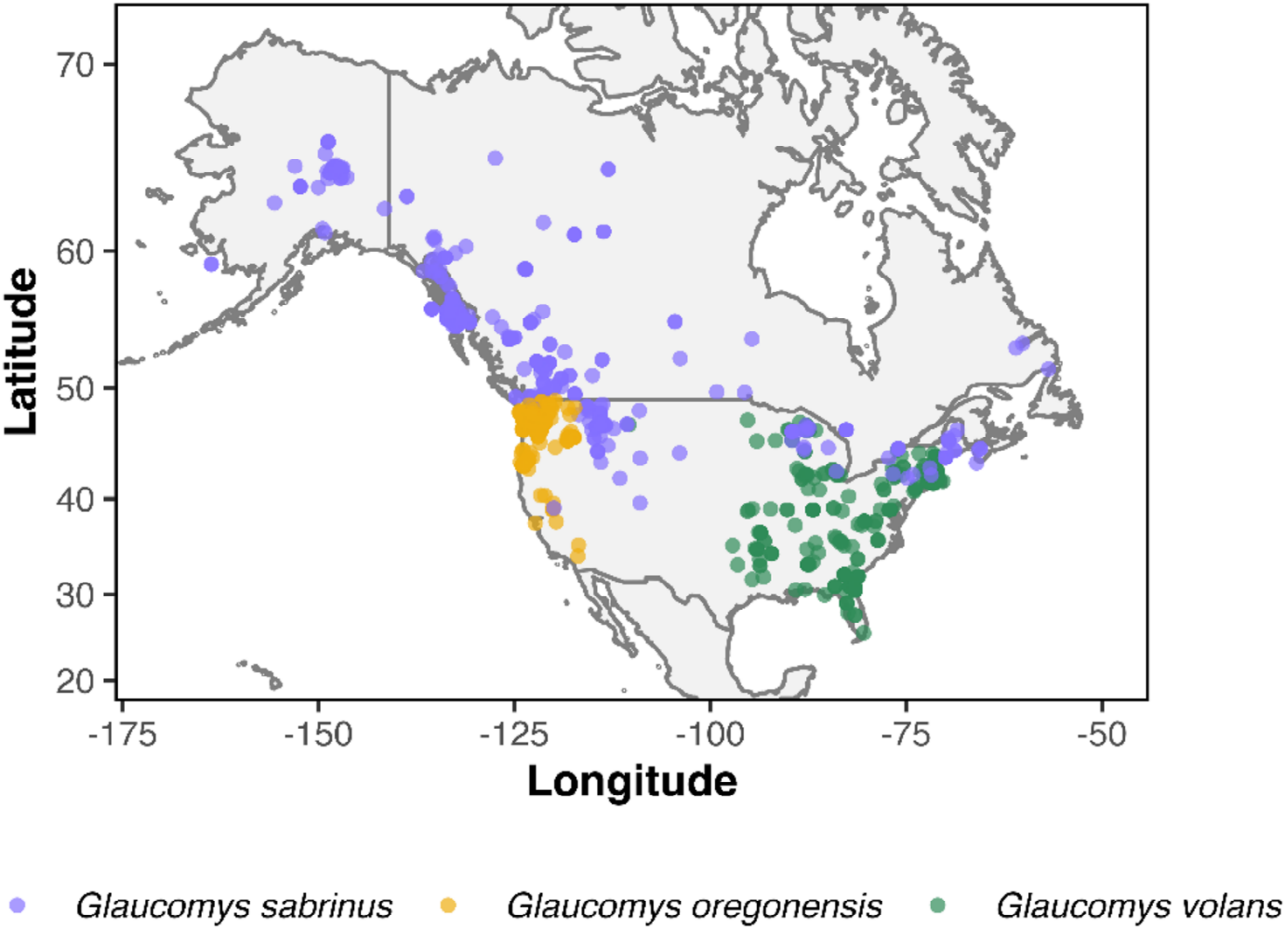
Map of *Glaucomys* specimen collection locations. Points represent individual specimens obtained from natural history collections across North America (*n* = 1649 specimens). Purple points represent *G. sabrinus* specimens (*n* = 814), orange points represent *G. oregonensis* specimens (*n* = 475), and green points represent *G. volans* specimens (*n* = 360).

## 3 Results

Across all species, all morphological traits had a significant relationship (*p <* 0.03) with latitude. Consistent with Bergmann's Rule, both weight (*y =* 2.823*x−*24.768, *R* ^2^ = 0.028, *p <* 0.001) and total body length (*y =* 2.879*x +* 143.014, *R* ^2^ = 0.331, *p* < 0.001) increased with increasing latitude (Figure 2). Ear length and tail length increased with latitude (ear length: *y =* 0.30*x +* 20.976, *R* ^2^ = 0.003, *p =* 0.028; tail length: *y* = 1.373*x* + 59.654, *R* ^2^ = 0.310, *p* < 0.001), contradicting the prediction that appendages become shorter in colder environments (Figure 3).

**FIGURE 3:**
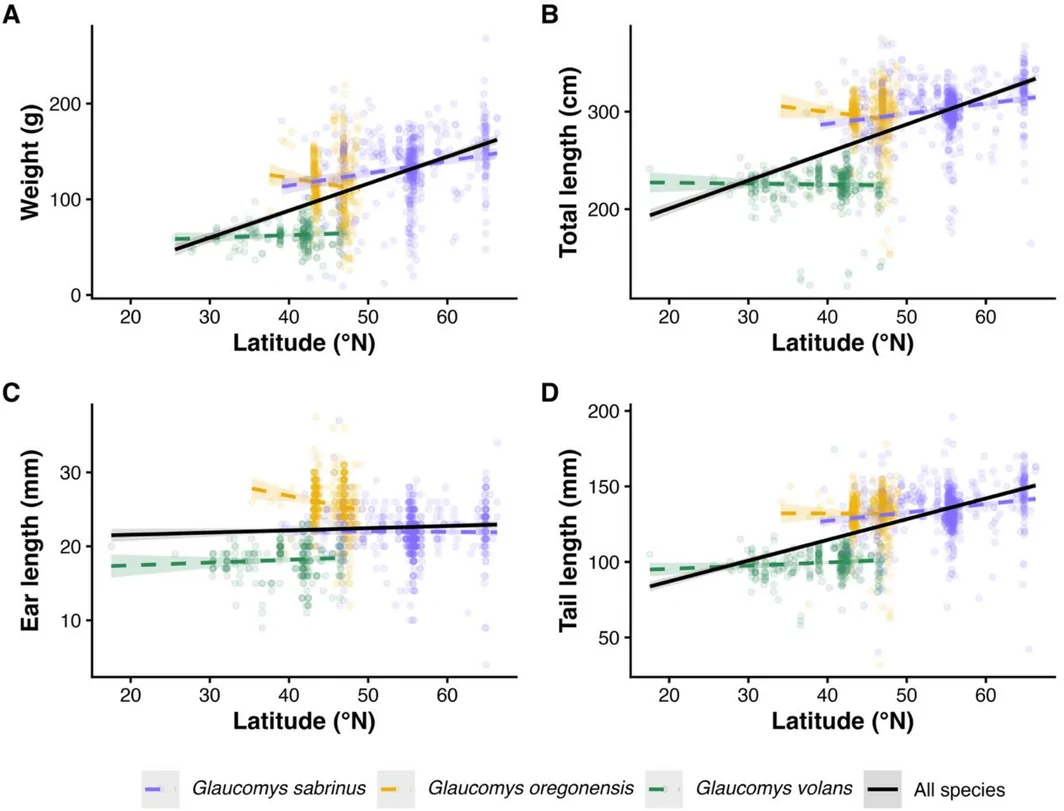
Tests of Bergmann's Rule (A, B) and Allen's (C, D) rules across North American *Glaucomys*. Linear regression plots show relationships between latitude and (A) body weight, (B) total body length, (C) ear length, and (D) tail length. Color denotes species of *Glaucomys*. Each point represents an individual museum specimen (*n* = 1202 for weight, *n* = 1622 for length, *n* = 1439 for ear length, *n* = 1622 for tail length). Solid black lines indicate linear regression fits across all specimens, regardless of species. Across all species, Bergmann's rule was supported (weight: *Y* = 2.82*x*−24.8, *R* ^2^ = 0.028, *p* < 0.001; total body length: *Y* = 2.88*x* + 143, *R* ^2^ = 0.331, *p* < 0.001), while Allen's rule was rejected (ear length: *Y* = 0.030*x* + 21, *R* ^2^ = 0.003, *p* = 0.028; tail length: *Y* = 1.37*x* + 59.7, *R* ^2^ = 0.310, *p* < 0.001). Colored dashed lines indicate linear regression fits corresponding to species, with shaded areas representing 95% confidence intervals. *G. sabrinus* largely followed the overall pattern, *G. oregonensis* exhibited decreasing ear length but no significant relationship between latitude and body size, and *G. volans* showed significant increases only in tail length with latitude.

*G. sabrinus* exhibited similar patterns to those observed across all species. Both weight (*y* = 1.288*x* + 62.775, *R* ^2^ = 0.046, *p* < 0.001) and total body length (*y* = 1.037*x* + 246.012, *R* ^2^ = 0.050, *p* < 0.001) increase with increasing latitude. Ear length showed no relationship with increasing latitude (*y* = −0.008*x* + 22.462, *R* ^2^ < 0.001, *p* = 0.699), whereas tail length significantly increased with increasing latitude (*y =* 0.554*x* + 105.039, *R* ^2^ = 0.044, *p* < 0.001) (Figure 3).

*G. oregonensis* exhibited a different pattern. Weight (*y =* −1.324*x* + 175.254, *R* ^2^ = 0.008, *p* = 0.078) and total length (*y* = −0.990*x* + 339.253, *R* ^2^ = 0.007, *p* = 0.074) both showed weak, nonsignificant declines with increasing latitudes, opposite to the prediction of Bergmann's rule. In contrast, ear length decreased with latitude (*y* = −0.213*x* + 35.355, *R* ^2^ = 0.019, *p* = 0.003), consistent with Allen's rule. Tail length showed no relationship with latitude (*y* = −0.010*x* + 132.555, *R* ^2^ < 0.001, *p* = 0.972) (Figure 3).

*G. volans* exhibited few significant relationships, other than tail length. Weight (*y* = 0.280*x* + 51.344, *R* ^2^ = 0.007, *p =* 0.208), total body length (*y* = −0.096*x* + 229.058, *R* ^2^ < 0.001, *p* = 0.683), and ear length (*y* = 0.038*x* + 16.668, *R* ^2^ = 0.004, *p* = 0.277) were not related to latitude. Tail length, however, increased with latitude (*y* = 0.211*x* + 91.214, *R* ^2^ = 0.012, *p* = 0.039), contrary to Allen's rule (Figure 3).

## 4 Discussion

Our findings indicate that North American flying squirrels (*Glaucomys* spp.) show mixed support for classic ecogeographical rules. Across all species, body size increased with latitude. This relationship was also significant within *G. sabrinus* , our most abundant and geographically widespread species, consistent with Bergmann's rule: individuals from higher latitudes were heavier and longer than those at lower latitudes. Previous studies have established that, compared to arboreal and terrestrial sciurids, flying squirrels have lower body mass relative to body size as a potential adaptation for gliding (Hayssen 2008). Our results extend this understanding by suggesting that selective pressures for latitudinal increase in weight may reflect indirect thermoregulatory advantages via reduced surface‐area‐to‐volume ratios at higher latitudes, consistent with reports from other sciurids and temperate small mammals (Brown and Lee 1969; Thorington and Heaney 1981). This observed latitudinal cline in body mass could represent a trade‐off between aerodynamic functioning to optimize gliding efficiency and “wing”‐loading while accounting for thermoregulatory constraints. *G. oregonensis* and *G. volans* both showed insignificant relationships with latitude, possible because of the smaller sample sizes and narrower geographic ranges of samples, and/or that these species may respond differently to environmental gradients than *G. sabrinus* .

Our results provide limited support for Allen's rule. Across all *Glaucomys* species, ear length increased slightly with latitude, contradicting the prediction that appendages become shorter in colder climates. However, the effect size was extremely small (*R* ^2^ = 0.003), indicating that latitude explains very little of the observed variation in ear morphology, even though the overall relationship was statistically significant. Species‐specific analyses further highlighted the inconsistency of this pattern. Neither *G. sabrinus* nor *G. volans* exhibited significant relationships between ear length and latitude independently, while *G. oregonensis* showed the opposite trend, with ear length decreasing toward higher latitudes. These contrasting results suggest that temperature alone is insufficient to explain variation in ear morphology within flying squirrels. Although mammalian ears can function as important structures for heat exchange, appendage size is also influenced by other ecological and functional pressures, which may weaken or obscure thermoregulatory patterns predicted by Allen's rule (Ryding et al. 2021). Furthermore, the negative relationship observed in *G. oregonensis* should be interpreted cautiously because of its comparatively smaller sample size and more restricted geographic distribution.

Tail length showed a much more consistent pattern across analyses, with longer tails occurring at higher latitudes and therefore opposing the predictions of Allen's rule and thermoregulatory predictions. This general discordance aligns with existing literature that suggests that tail morphology may be influenced more strongly by functional and ecological demands (Hayssen 2008; Thorington and Heaney 1981). This evidence aligns with Thorington and Heaney (1981) who found that tail length scales positively with body size across gliding sciurids, proposing that larger squirrels require longer tails to facilitate maneuverability during gliding. Thus, selective pressure for aerodynamic performance may outweigh thermoregulatory constraints in shaping tail length across latitudinal gradients.

While comparative studies across mammals often report longer tails in tropical species (Alhajeri et al. 2020; Alroy 2019; Ballinger and Nachman 2022), these patterns reflect broad taxonomic and functional comparisons. In contrast, our results highlight that within a specialized locomotor guild, such as gliding mammals, tail length may respond more strongly to biomechanical and ecological constraints than to ambient temperature alone. Together, these findings reinforce that appendage length does not universally track thermoregulatory predictions and that functionally critical traits may exhibit latitudinal patterns that directly oppose Allen's rule. Investigating the adherence to these thermoregulatory‐adapted ecogeographical rules, and the exceptions of them, is critical to understanding organismal responses in changing environments.

Although several traits exhibited significant latitudinal trends, considerable variation remained across species and among individuals. This variability likely reflects the influence of multiple ecological and functional factors compounded by the temporal breadth of our dataset, which spans more than a century. Due to our specimens spanning more than a century, morphological variation likely reflects not only spatial differences across latitude but also temporal changes associated with environmental change. Temporal changes in morphological traits are likely influenced by climatic change and habitat modification (Millien et al. 2006). Increasing habitat fragmentation in recent decades has affected the distribution and persistence of *Glaucomys* species (Holloway et al. 2012), which may in turn influence body size and morphology. While this dataset does not allow for robust statistical analyses, investigating climate‐driven changes to morphological traits over a vast timescale would provide insight to anthropogenic impacts on microevolutionary dynamics and a deeper understanding of the adaptive capacity of flying squirrels under environmental change.

## Author Contributions

**Tiffany A. Paalman:** conceptualization (lead), data curation (lead), formal analysis (equal), validation (equal), visualization (equal), writing – original draft (lead), writing – review and editing (lead). **Joseph P. Morton:** formal analysis (equal), visualization (equal), writing – review and editing (equal). **Cori Lopazanski:** formal analysis (supporting), visualization (supporting), writing – review and editing (equal). **Brian R. Silliman:** supervision (equal), writing – review and editing (equal). **Amy T. Wolf:** conceptualization (equal), data curation (supporting), supervision (equal), writing – review and editing (equal).

## Funding

This work received no specific funding from an agency in the public, commercial, or nonprofit sector. Tuition and stipend support for TA Paalman while developing this manuscript was provided by Duke University through the Nicholas School of the Environment.

## Conflicts of Interest

The authors declare no conflicts of interest.

## Data Availability

The data and code that support the findings of this study are provided in the Duke Data Repository at https://doi.org/10.7924/r4r550, as well as in flying‐squirrels‐ecogeo at https://github.com/tiffanypaalman/flying‐squirrels‐ecogeo.
